# Psychosocial Risk in COVID Context: The Impact of Economic Factors and Labour Protection Policy (ERTEs) in Spain

**DOI:** 10.3390/ijerph20031824

**Published:** 2023-01-19

**Authors:** Enrique Iglesias Martínez, Pablo Yáñez Legaspi, Esteban Agulló-Tomás, José Antonio Llosa

**Affiliations:** 1 Department of Social Education, Padre Ossó Faculty, University of Oviedo, 33008 Oviedo, Spain; 2 Faculty of Juridic Sciences, University Rovira i Virgili, 43002 Tarragona, Spain; 3 Department of Psychology, University of Oviedo, 33003 Oviedo, Spain

**Keywords:** working poverty, anxiety, syndemic, confinement, social risk factors, work, ERTEs

## Abstract

The pandemic and the current situation have caused working poverty and therefore social risk, which implies a deterioration in well-being, affecting mental health and anxiety. In this context, the employment situation tends to be regarded ignoring previous social differences, economic and mental components, which should be considered when establishing priorities to program a global action of various synergistic elements. The study involved 4686 people (3500 women and 1186 men). They all completed a questionnaire that evaluated their anxiety, employment situation, income, changes of working status, and fears of becoming infected at the workplace. The results show the need to take into account the social determinants of mental health in vulnerable groups due to socioeconomic factors, job changes, contractual changes, age, or gender, considering the need to generate strategies to manage mental health and deal with it at a structural level, therefore displacing individual focus policies and interventions. An example of these policies are ERTEs (record of temporary employment regulation), constituting a perceived measure of protection and acting as an effective buffer against the economic crisis, thus reducing anxiety.

## 1. Introduction

The social determinants of health (SDH) are defined as the circumstances in which people are born, grow up, work, live, and age, including the broader set of forces and systems that influence the conditions of everyday life [1,2,3]. The COVID-19 pandemic has spread in a context of social and economic inequalities that become social determinants affecting health [4,5] and resulting in a syndemic; i.e., the prevalence and severity cannot be reduced to any one of the many variables involved in it, suggesting a global composite with several synergistic elements, including viral disease, social differences, economic and mental components, that should be considered when setting priorities for programming action [6,7,8], especially with vulnerable populations [9,10,11].

It should be noted that adverse psychological circumstances (stress and anxiety) increase susceptibility to illness, which influences the onset, course, and outcome of infectious diseases [12,13]. Within the group of infectious diseases is COVID-19, whose direct and indirect psychological and social effects are pervasive and could affect mental health [14,15]. Social determinants make people with lower incomes more vulnerable to infection even when they have no previous health problems, as psychological stress and anxiety resulting from low or even no income is associated with immunosuppression [16,17] and infectious diseases [18]. Factors influencing this include living conditions, poverty, and uncertainty about the future (i.e., risk of unemployment) as well as social support [19,20,21]. Similarly, sharing this scenario induces perceptions of fear and anxiety that, sustained over time, become chronic and damaging to health [22,23]. As the global economy continues to be affected, people will continue to live in fear and anxiety. The impact of the current pandemic on the incidence and severity of disorders related to these two variables will therefore be very heterogeneous. Fear is an emotional response to a real or perceived imminent threat, while anxiety is the anticipation of a future threat [24]. To reduce the uncertainty about the job future, the Spanish government has implemented ERTEs (record of temporary employment regulation) as a response to the economic impact of the COVID-19 pandemic. ERTEs are a financial instrument. They allows companies to temporarily suspend or reduce the working hours of employees due to economic, technical, organizational, or production reasons. This measure is intended to help companies mitigate the impacts of a crisis or recession on their workforce while at the same time providing benefits to the workers so that they are not deprived of their income; they thus receive a significant part of their normal earnings from the state social security funds. The Spanish government often encourages or requires companies to use ERTEs as a way to avoid layoffs and protect jobs during difficult economic times. 

On the other hand, in addition to the above, social distancing is a necessary health measure to reduce the spread of COVID-19 [25]. The scientific literature has shown that this norm has become an important anxiogenic factor [26,27]. Knowing the psychological and social consequences of the confinement process is of great interest, as the possible benefits of compulsory mass quarantine entail great costs at the psychological level [28,29,30] as well as exacerbating economic and labour crises in the population that negatively affect mental health [31].

In this respect, this study aims to test whether COVID-19 acts as a socially neutral disease, i.e., whether it affects everyone equally and in the same way, or whether there are socio-economic factors that influence the psychological state of the person, affecting susceptibility to it. The interactions of these factors are important for prognosis, treatment, and health policies. To this end, three hypotheses are designed to be tested. The first hypothesis is that the pandemic does not affect everyone equally and that there may be some impact on mental health related to social determinants of health (Hypothesis 1). Knowing the essential role of income level, it is hypothesised that the higher the income level, the lower the anxiety levels (Hypothesis 2). Thirdly, it is hypothesised that perceived state-driven measures of protection and effective buffering against the economic crisis are anxiety-reducing (Hypothesis 3).

## 2. Materials and Methods

### 2.1. Participants

A total of 4686 participants (3500 men and 1186 women) between 12 and 72 years old (M = 37.90 years, S.D = 12.78 years) participated in this research. The subjects of this study were in a situation of confinement when answering the questionnaire.

### 2.2. Procedure

The collection of data was carried out in digital format through different universities, social networks, and other communication platforms. The questionnaire was sent throughout the Spanish territory. The purpose was to collect data for the period during which the confinement of the population was stricter in Spain, when leisure was limited to staying indoors. In this period, the economy was paralyzed, and people’s uncertainty about the future was greater. Their anonymity was respected, and data confidentiality was guaranteed. Consequently, they were not asked for identification variables. This research was conducted in accordance with the protocol of Ethical Committee of the University of Oviedo and the Declaration of Helsinki.

### 2.3. Analysis Vein

The collected data were recorded and analysed using the statistical software R (R Development Core Team), version 3.6.0. Bivariate correlation analysis, descriptive analysis, quantitative analysis, and multivariate linear model analysis were performed to determine the influence of confinement on respondents’ anxiety. Descriptive analysis was performed providing relative and absolute frequency distributions for qualitative variables and measurements of position and dispersion for quantitative variables. Relationships between qualitative variables were assessed with Pearson’s chi-square test and Fisher’s test, depending on whether or not the hypothesis on expected frequencies was verified. When quantitative variables were compared between two groups, Student’s *t*-test was used for independent samples with Welch’s correction for different variances. If the groups to be compared were three or more in number, the ANOVA test with Tukey’s post hoc test was used. A multivariate linear model was constructed to determine the factors associated with a greater difference between state and trait anxiety, and a binary logistic model was constructed to predict the probability of presenting state anxiety. For the variable defined as the difference between trait and state anxiety, the factors associated with it were studied by means of a multivariate linear model. The level of significance used was 0.05. 

### 2.4. Instruments

A record sheet was drawn up that collected information about socio-demographic variables through a list in which all the answers that the person considered appropriate could be marked. This battery of questionnaires measures employment status (salary, employment situation, contractual change*),* stressful job, sex, and age.

To collect the anxiety variable in this study, the State–Trait Anxiety Inventory (STAI) [32] in its Spanish adaptation [33] was used. The adapted version consists of 40 items divided in two groups. The first part (S/A) assesses a transient emotional state characterized by subjective feelings that are consciously perceived. The second (T/A) indicates a relatively stable anxious propensity that characterises individuals with a tendency to perceive situations as threatening. The application time is approximately 20 min. As for the psychometric guarantees of the instrument, it has a good internal consistency with a Cronbach’s alpha of 0.93 for the state scale and 0.87 for the trait scale.

## 3. Results

### 3.1. Descriptive Statistics 

Regarding the variable “Do you have a job?”, of the 4686 people who participated in the study, 68.54% said that they had a job, and 31.46% said that they did not have a job (not considering being exclusively a student as a job). In relation to the variable “Employment status”, the following frequency distribution was obtained: employed (42.25%), student (16.26%), civil servant (13.85%), unemployed (10.29%), self-employed (7.36%), other (4.57%), retired (2.9%), and domestic tasks (2.52%). As for the variable “monthly salary”, the distribution of the sample is as follows: no income (20.64%), less than 500 euros (7.98%), between 500 and 800 euros (9.54%), between 800 and 1200 euros (23.28%), between 1200 euros and 1600 euros (18.99%), and more than 1600 euros (19.57%).

On the variable “Have you undergone a contractual change?”, 77.04% had not undergone any change; other responses included ERTE (13.02%), enforced holidays (5.61%), dismissal (3.82%), and salary increase (0.51%). Regarding the variable “Do you have to leave home to go to work?”, 76.57% answered no, and 23.43% answered affirmatively. Finally, to the variable “Does going to work mean a stressful situation for fear of contagion?”, answers included “no” (20.29%), “I don’t work” (39.2%), “I work online” (23.9%), and “yes” (16.6%).

### 3.2. Multivariate Binary Logistic Binary Model

To jointly assess all possible factors associated with the presence of *state anxiety*, a binary logistic model was constructed (Table 1). The full model with all collected variables was simplified using a stepwise selection method, obtaining a significant model according to the likelihood ratio test (*p* < 0.001), with a Nagelkerke R2 = 19%.

Observing the odds ratio (OR) values in the simplified model table, it can be seen that if one is younger (differences between all age groups with respect to being over the age of 55, except for ages 36–55, which are not observed (*p* = 0.607), having a job is also associated with a higher risk of presenting anxiety. In terms of *employment status*, the self-employed and students carry a higher risk of anxiety than employees, as do being unemployed or engaged in domestic tasks. In terms of *salary*, although some coefficients are at the borderline of significance, the higher the salary, the lower the risk of anxiety. Having a *stressful job due to fear of contagion, teleworking, or not working* also present a higher risk than individuals who claim *not to have a stressful job due to fear of contagion.*

Likewise, analysis was carried out to study the relationship between *monthly salary and gender*, obtaining that there is an association (Pearson’s chi-square test, *p*-value < 0.001) (Table 2).

Similarly, “*Have you undergone a contractual change?*” is also associated with *state anxiety*, (Pearson’s chi-square test, *p*-value = 0.045) (Table 3).

### 3.3. Multivariate Linear Model

In the case of the difference between *state and trait anxiety*, given its quantitative nature, a multivariate linear model was constructed including all the variables collected as predictors. Subsequently, a stepwise selection algorithm was applied, resulting in the following model shown in the table, with the coefficients and associated significance. The linear fit was significant (*p* < 0.001), but the explanatory power was poor (R2_adj = 6.22%). 

Firstly, the state–trait anxiety difference variable differs according to the different levels of the variable “Do you have a job?”. Considering the sufficient sample size and the rejection of the hypothesis of equality of the two population variances (*p*-value = 0.024), we obtained that the hypothesis of equality of means is rejected (Welch’s test, *p*-value < 0.001) (Table 4).

As it is a sufficient sample size, and the hypothesis of homogeneity of variances is rejected (Bartlett’s test, *p*-value < 0.001), the hypothesis that the averages are equal is rejected (Kruskal–Wallis test, *p*-value < 0.001). Dunn’s test indicated that in certain pairs of levels, there are significant differences between them. In order of significance, the following relationships resulted: retired and salaried (*p*-value < 0.001), retired and civil servant (*p*-value < 0.001), retired and self-employed (*p*-value < 0.001), other and retired (*p*-value < 0.002), student and salaried (*p*-value = 0.014), retired and student (*p*-value = 0.017), and unemployed and retired (*p*-value = 0.031), respectively. In contrast, there are no differences between the other levels. 

The studies were then repeated for the difference variables state–trait anxiety and “Have you undergone a contractual change?” (Table 5).

As there is a sufficient sample size, the hypothesis of normality is not rejected in all modalities (Shapiro–Wilk test, salary increase, *p*-value = 0.835), and the hypothesis of homogeneity of variances is rejected (Bartlett’s test, *p*-value = 0.017), while the hypothesis that the averages are equal is not rejected (Kruskal–Wallis test, *p*-value = 0.146).

Considering the sufficient sample size and that the hypothesis of equality of the two population variances is not rejected (F-test of variances, *p*-value = 0.623), it is observed that the hypothesis of equality of population means is rejected (Student’s *t*-test, *p*-value < 0.001).

## 4. Discussion

This work shows that the pandemic does not affect everyone equally, as there are certain inequalities in mental health related to social determinants, which leads us to conclude that we are experiencing a syndemic. This result confirms the first hypothesis of the study and is in line with the work of Horton [34] or previous studies such as Kwan and Ernst [35], which pointed out that infectious diseases have a high potential to produce syndemic behaviour, as infections of this type develop in environments of health inequality, economic inequality, stress, or the structural underdevelopment of a country. These social factors will feed on each other and end up interacting to cause complex aftereffects, as is hinted in our study. Thus, we agree with Mendenhall’s proposal [10] that the measures to be taken in the face of this type of crisis should have an approach that mitigates the social consequences of pandemics that affect people’s general health, especially those who may be most vulnerable. In this line, global public health managers can, through clinical practice and community interventions, recognise the negative social factors that influence the acquisition of disease as well as increased morbidity and mortality in the most disadvantaged populations. 

Likewise, our work coincides with previous approaches such as those of Li [36], verifying the existence of certain levels of anxiety in the face of the health crisis, where the most reported psychological consequences are depression, anxiety, and the symptoms derived from this. Among the factors correlated with anxiety in this study is the level of income, confirming the second hypothesis put forward in the study. Subjects with lower salaries or those who report having no salary have higher levels of anxiety than, for example, those who admit to earning more than EUR 1600. It seems clear that income acts as a factor influencing anxiety levels, with a particular emphasis on women, who on average have lower income than men and higher anxiety scores. These findings coincide with the results found by Iglesias Martínez et al. [37] and Wang [38], in which they stated that financial situation is one of the factors that explain why women had three times higher anxiety levels than men during the pandemic. Therefore, economic inequalities seem to act as a synergistic factor affecting this population, which will require the development of specific conceptual frameworks in order to implement prevention and control programmes to address comorbidities [34,39]. The data from the study, therefore, put us in agreement with authors such as Gravlee [5], Khazanchi [40], and Laster Pirtle [41], who highlighted the importance of structural conditions that determine higher morbidity and mortality from COVID-19 in communities already suffering from poverty, inequality, and social exclusion.

On the other hand, young people (students) as well as the self-employed are more at risk of anxiety. The former lack income, and the latter are directly affected by the economic consequences of the pandemic. It should be noted that the pandemic has acted as a trigger for anxiety mainly in the young population [27,42]. On the other hand, people who are obliged to travel to work have a stressful job because they are exposed to a possible infection, and similarly, evidence shows that those who work online are also affected by anxiety. In the first two cases, concern about possible infection with the virus seems to be the driving force behind the anxiety. In the case of online work, the forced adaptation to the new situation may have played a determining role, as only those who have a non-stressful job due to exposure to the disease and have not undergone significant changes have lower levels of anxiety. With respect to the employment situation, it is observed that retired people and civil servants are the ones who show the least increase in anxiety in the face of the crisis and show lower average state anxiety levels, mainly due to a perception of greater economic security and stability compared to the self-employed, students, the unemployed, and people who take care of domestic tasks, whose financial situation is perceived as unstable and uncertain in the face of any crisis. In short, as Laster Pirtle [41] and Tokuda [43] argued, the poor development of job security in these contexts leads to a deterioration in general well-being, with poverty being a factor that negatively affects health.

Finally, the restrictions resulting from the pandemic have had a major impact on employment and have led to greater inequality in employment [37,40]. Among the consequences are contractual changes. Thus, we can observe in this study that people who have not undergone any change in their contract or have seen their salary increased have low levels of anxiety compared to people who have been forced to take holidays or have been made redundant, followed by people with temporary lay-offs (ERTEs). This result is a contribution of our own that helps to increase the knowledge that is being gained about this phenomenon, with these ERTEs constituting a perceived measure of protection and acting as an effective buffer against the economic crisis, thus reducing anxiety. This result confirms the third hypothesis of our work: that the perceived measures of protection and effective buffering against the economic crisis promoted by the state have a negative effect on the increase of anxiety. 

## 5. Conclusions

These findings allow for a critical evaluation of activation policies that only have an individual focus, the alternative of which proposes strengthening interventions that foster social support, particularly among people with lower incomes or who are more exposed to economic and/or employment crises, as Llosa already noted [44]. In this respect, it is necessary, as authors such as Broding [45] and Hyland [46] pointed out, for the state to guarantee and reinforce minimum conditions of security and certainty in the face of possible future crises since, as in this study, a deterioration of mental health has been observed indirectly as a consequence of the costs associated with worrying about the economic and social situation. Furthermore, in accordance with Bambra [47], public health policies must ensure in the long term that pandemics do not increase social inequalities for future generations while taking into account their impact on mental health. It is therefore crucial to be aware of anxiety and stress management at a societal level. As the population groups with lower socio-economic status are more prone to negative outcomes related to these, they can benefit from targeted short- and long-term interventions that provide adequate access to health and economic infrastructure, stimulating communities to organise themselves, which is in agreement with authors such as Lazzarino [48]. Finally, in order to determine to what extent the pandemic has affected particular groups of society, it would be necessary to carry out surveys that would be representative of them.

## Figures and Tables

**Table 1:** Factors associated with the presence of state anxiety.

	Coef	*p*	OR	IC 95−	IC 95+
Ages 18 to 25 years	0.794	0.002	2.213	1.329	3.691
Ages 26 to 35 years	0.566	0.006	1.762	1.173	2.641
Ages 36 to 45 years	0.477	0.016	1.611	1.093	2.368
Ages 46 to 55 years	0.105	0.607	1.111	0.743	1.658
Employment sit.: Employed	0.597	0.030	1.816	1.065	3.129
Employment sit.: Self-employed	−0.388	0.041	0.679	0.470	0.988
Employment sit.: Student	0.896	0.006	2.449	1.312	4.657
Employment sit.: Civil servant	−0.148	0.335	0.863	0.639	1.166
Employment sit.: Retired	−0.030	0.935	0.971	0.473	2.004
Employment sit.: Others	0.098	0.702	1.103	0.676	1.844
Employment sit.: Unemployed	0.610	0.045	1.841	1.021	3.375
Employment sit.: Domestic tasks	0.895	0.046	2.447	1.041	6.097
Salary: No income	0.517	0.056	1.678	0.987	2.848
Salary: Less than EUR 500	0.449	0.059	1.567	0.986	2.513
Salary: Between EUR 500 and EUR 800	0.667	0.003	1.947	1.265	3.036
Salary: Between EUR 800 and EUR 1200	0.388	0.014	1.474	1.081	2.013
Salary: Between EUR 1200 and EUR 1600	0.315	0.032	1.370	1.028	1.828
Stressful job: Unemployed	0.376	0.007	1.456	1.107	1.914
Stressful job: Employed	1.700	<0.001	5.476	3.754	8.137
Stressful job: Teleworking	0.486	<0.001	1.626	1.251	2.116

**Table 2:** Monthly salary and gender.

		Men			Women	
	*n*	%Col	%Row	*n*	%Col	%Row
No income	218	18.37	22.54	749	21.41	77.46
Less than EUR 500	78	6.57	20.86	296	8.46	79.14
Between EUR 500 and EUR 800	62	5.22	13.87	385	11.00	86.13
Between EUR 800 and EUR 1200	211	17.78	19.34	880	25.15	80.66
Between EUR 1200 and EUR 1600	258	21.74	28.99	632	18.06	71.01
More than EUR 1600	360	30.33	39.26	557	15.92	60.74

**Table 3:** “Have you undergone a contractual change?” is also associated with state anxiety.

		Absence			Presence	
	*n*	%Col	%Row	*n*	%Col	%Row
No	933	79.27	25.84	2677	76.29	74.16
Fired	41	3.48	22.91	138	3.93	77.09
ERTEs ^1^	152	12.91	24.92	458	13.05	75.08
Forced vacation	47	3.99	17.87	216	6.16	82.13
Salary increase	4	0.34	16.67	20	0.57	83.33

^1^ None; record of temporary employment regulation (ERTEs, acronym in Spanish).

**Table 4:** Difference between state and trait anxiety, “Do you have a job?”, and employment situation.

	*n*	Mean	Median	D.t	P25	P75
No	1474	5.63	5.00	10.54	−1.00	12.00
Yes	3212	7.37	7.00	10.03	1.00	14.00
Employees	1980	7.38	8.00	9.97	1.00	14.00
Self-employed	345	7.33	7.00	10.28	1.00	14.00
Student	762	6.05	6.00	11.59	−1.00	12.00
Civil servant	649	7.12	7.00	9.80	1.00	13.00
Retired	136	3.55	3.00	7.89	−2.00	8.00
Others	214	7.23	7.00	10.53	1.25	13.00
Unemployed	482	5.94	6.00	9.75	−0.75	12.00
Domestic tasks	118	5.92	6.00	9.95	−0.75	12.00

**Table 5:** Difference between state and trait anxiety and “Have you undergone a contractual change?”.

	*n*	Mean	Median	D.t	P25	P75
No	3610	6.85	7.00	10.31	0.00	13.00
Fired	179	6.47	7.00	10.00	0.00	11.00
ERTE ^1^	610	6.19	6.00	9.44	0.00	13.00
Forced vacation	263	7.83	8.00	11.00	1.50	13.50
Salary increase	24	9.79	10.50	8.63	5.00	13.25

^1^ None; record of temporary employment regulation (ERTEs, acronym in Spanish).

## Data Availability

Data available on request due to privacy restrictions.

## References

[1] Benach J., Vives A., Amable M., Vanroelen C., Tarafa G., Muntaner C. (2014). Precarious Employment: Understanding an Emerging Social Determinant of Health. Annu. Rev. Public Health.

[2] Head J., Chandola T., Benach J., Muntaner C., Santana V. (2007). Psychosocial working conditions and social inequalities in health. Final Report to the WHO Commission on Social Determinants of Health (CSDH) Employment Conditions Knowledge Network.

[3] World Health Organization (2008). Closing the Gap in a Generation.

[4] Ahmad A., Mueller C., Tsamakis K. (2020). COVID-19 PANDEMIC COVID-19 Pandemic: A Public and Global Mental Health Opportunity for Social Transformation?. BMJ Br. Med. J..

[5] Gravlee C.C. (2020). Systemic Racism, Chronic Health Inequities, and COVID-19: A Syndemic in the Making?. Am. J. Hum. Biol..

[6] Singer M., Bulled N., Ostrach B. (2012). Syndemics and Human Health: Implications for Prevention and Intervention. Ann. Anthropol. Pract..

[7] Singer M., Bulled N., Ostrach B., Mendenhall E. (2017). Syndemics and the Biosocial Conception of Health. Lancet.

[8] Smith J.A., Judd J. (2020). COVID-19: Vulnerability and the Power of Privilege in a Pandemic. Health Promot. J. Aust..

[9] Llosa J.A., Agulló-Tomás E. (2022). Technodiscipline of Work: Does Post-Pandemic Platform Employment Generate New Psychosocial Risks?. Int. J. Environ. Res. Public. Health.

[10] Mendenhall E., Kohrt B.A., Norris S.A., Ndetei D., Prabhakaran D. (2017). Non-communicable disease syndemics: Poverty, depression, and diabetes among low-income populations. Lancet.

[11] Vancea M., Utzet M. (2017). How unemployment and precarious employment affect the health of young people: A scoping study on social determinants. Scand. J. Public Health.

[12] Huang Y., Zhao N. (2020). Generalized Anxiety Disorder, Depressive Symptoms and Sleep Quality during COVID-19 Outbreak in China: A Web-Based Cross-Sectional Survey. Psychiatry Res..

[13] Hao F., Tan W., Jiang L., Zhang L., Zhao X., Zou Y., Hu Y., Luo X., Jiang X., McIntyre R.S. (2020). Do psychiatric patients experience more psychiatric symptoms during COVID-19 pandemic and lockdown? A case-control study with service and research implications for immunopsychiatry. Brain Behav. Immun..

[14] Holmes E.A., O’Connor R.C., Perry V.H., Tracey I., Wessely S., Arseneault L., Ballard C., Christensen H., Silver R.C., Everall I. (2020). Multidisciplinary Research Priorities for the COVID-19 Pandemic: A Call for Action for Mental Health Science. Lancet Psychiatry.

[15] Vahia I.V., Blazer D.G., Smith G.S., Karp J.F., Steffens D.C., Forester B.P., Tampi R., Agronin M., Jeste D.V., Reynolds C.F. (2020). COVID-19, Mental Health and Aging: A Need for New Knowledge to Bridge Science and Service. Am. J. Geriatr. Psychiatry.

[16] Arévalo N.S.A., Briceño I.L., Cruz L.D.R., Rojas M.Á.B. (2021). Vulnerabilidad de las mujeres indígenas de la península de Yucatán frente a la pandemia COVID-19. Cienc. Intercult..

[17] Segerstrom S.C., Miller G.E. (2004). Psychological Stress and the Human Immune System: A Meta-Analytic Study of 30 Years of Inquiry. Psychol. Bull..

[18] Matilla-Santander N., González-Marrón A., Martín-Sánchez J.C., Lidón-Moyano C., Hueso À.C., Martínez-Sánchez J.M. (2019). Precarious employment and health-related outcomes in the European Union: A cross-sectional study. Crit. Public Health.

[19] Moon J.Y., Kang S.J. (2020). Study on factors affecting depression of working poor-Hierarchical regression analysis of income, health, housing, labor and drinking factors by gender. Stud. Life Cult..

[20] Southwick S.M., Charney D.S. (2012). The Science of Resilience: Implications for the Prevention and Treatment of Depression. Science.

[21] Jiang L., Lavaysse L.M. (2018). Cognitive and Affective Job Insecurity: A Meta-Analysis and a Primary Study. J. Manag..

[22] Slagboom M.N., Reis R., Tsai A.C., Büchner F.L., van Dijk D.J.A., Crone M.R. (2021). Psychological Distress, Cardiometabolic Diseases and Musculoskeletal Pain: A Cross-Sectional, Population-Based Study of Syndemic Ill Health in a Dutch Fishing Village. J. Glob. Health.

[23] Whitehead M., Pennington A., Orton L., Nayak S., Petticrew M., Sowden A., White M. (2016). How Could Differences in ‘Control over Destiny’ Lead to Socio-Economic Inequalities in Health? A Synthesis of Theories and Pathways in the Living Environment. Health Place.

[24] American Psychiatric Association (2013). Diagnostic and Statistical Manual of Mental Disorders: DSM-5TM.

[25] Center for Disease Prevention and Control [CDC] (2020). Coping with Stress.

[26] Barbisch D., Koenig K.L., Shih F.-Y. (2015). Is There a Case for Quarantine? Perspectives from SARS to Ebola. Disaster Med. Public Health Prep..

[27] Iglesias-Martínez E., Roces-García J., Bermúdez-Rey M.T. (2021). Study on Regular Habits during Confinement Periods and Their Influence on Anxiety (Estudio Sobre Los Hábitos Regulares En Periodos de Confinamiento y Su Influencia En La Ansiedad). Stud. Psychol..

[28] Bai Y., Lin C.-C., Lin C.-Y., Chen J.-Y., Chue C.-M., Chou P. (2004). Survey of Stress Reactions Among Health Care Workers Involved With the SARS Outbreak. Psychiatr. Serv..

[29] Rubin G.J., Wessely S. (2020). The Psychological Effects of Quarantining a City. BMJ.

[30] Wu P., Fang Y., Guan Z., Fan B., Kong J., Yao Z., Liu X., Fuller C.J., Susser E., Lu J. (2009). The Psychological Impact of the SARS Epidemic on Hospital Employees in China: Exposure, Risk Perception, and Altruistic Acceptance of Risk. Can. J. Psychiatry.

[31] Brooks S.K., Webster R.K., Smith L.E., Woodland L., Wessely S., Greenberg N., Rubin G.J. (2020). The Psychological Impact of Quarantine and How to Reduce It: Rapid Review of the Evidence. Lancet.

[32] Spielberger C.D., Gorsuch R.L., Lushene R.E. (1999). STAI. Cuestionario de Ansiedad Estado/Rasgo.

[33] Buela-Casal G., Guillén-Riquelme A., Seisdedos N. (2011). STAI: Cuestionario de Ansiedad Estado-Rasgo. Adaptación Española.

[34] Horton R. (2020). Offline: COVID-19 Is Not a Pandemic. Lancet.

[35] Kwan C.K., Ernst J.D. (2011). HIV and Tuberculosis: A Deadly Human Syndemic. Clin. Microbiol. Rev..

[36] Li S., Wang Y., Xue J., Zhao N., Zhu T. (2020). The Impact of COVID-19 Epidemic Declaration on Psychological Consequences: A Study on Active Weibo Users. Int. J. Environ. Res. Public. Health.

[37] Iglesias Martínez E., Roces García J., Jiménez Arberas E., Llosa J.A. (2022). Difference between Impacts of COVID-19 on Women and Men’s Psychological, Social, Vulnerable Work Situations, and Economic Well-Being. Int. J. Environ. Res. Public. Health.

[38] Wang B., Li R., Lu Z., Huang Y. (2020). Does Comorbidity Increase the Risk of Patients with COVID-19: Evidence from Meta-Analysis. Aging.

[39] Manderson L. (2012). Review: Introduction to Syndemics: A Critical Systems Approach to Public and Community Health.

[40] Khazanchi R., Beiter E.R., Gondi S., Beckman A.L., Bilinski A., Ganguli I. (2020). County-Level Association of Social Vulnerability with COVID-19 Cases and Deaths in the USA. J. Gen. Intern. Med..

[41] Laster Pirtle W.N. (2020). Racial Capitalism: A Fundamental Cause of Novel Coronavirus (COVID-19) Pandemic Inequities in the United States. Health Educ. Behav..

[42] Losada-Baltar A., Márquez-González M., Jiménez-Gonzalo L., Pedroso-Chaparro M.D.S., Gallego-Alberto L., Fernandes-Pires J. (2020). Diferencias en función de la edad y la autopercepción del envejecimiento en ansiedad, tristeza, soledad y sintomatología comórbida ansioso-depresiva durante el confinamiento por la COVID-19. Rev. Esp. Geriatr. Gerontol..

[43] Tokuda Y., Ohde S., Takahashi O., Hinohara S., Fukui T., Inoguchi T., Butler J.P., Ueda S. (2009). Influence of Income on Health Status and Healthcare Utilization in Working Adults: An Illustration of Health among the Working Poor in Japan. Jpn. J. Political Sci..

[44] Llosa J.A., Agulló-Tomás E., Menéndez-Espina S., Rivero-Díaz M.L., Iglesias-Martínez E. (2022). Self-Criticism in In-Work Poverty: The Mediating Role of Social Support in the Era of Flexibility. Int. J. Environ. Res. Public Health.

[45] Broding H.C., Weber A., Glatz A., Buenger J. (2010). Working Poor in Germany: Dimensions of the Problem and Repercussions for the Health-Care System. J. Public Health Policy.

[46] Hyland P., Shevlin M., McBride O., Murphy J., Karatzias T., Bentall R.P., Martinez A., Vallières F. (2020). Anxiety and Depression in the Republic of Ireland during the COVID-19 Pandemic. Acta Psychiatr. Scand..

[47] Bambra C., Riordan R., Ford J., Matthews F. (2020). The COVID-19 Pandemic and Health Inequalities. J. Epidemiol. Community Health.

[48] Lazzarino A.I., Yiengprugsawan V., Seubsman S., Steptoe A., Sleigh A.C. (2014). The Associations between Unhealthy Behaviours, Mental Stress, and Low Socio-Economic Status in an International Comparison of Representative Samples from Thailand and England. Glob. Health.

